# Poor Dietary Guidelines Compliance among Low-Income Women Eligible for Supplemental Nutrition Assistance Program-Education (SNAP-Ed)

**DOI:** 10.3390/nu10030327

**Published:** 2018-03-08

**Authors:** Shinyoung Jun, Sowmyanarayanan V. Thuppal, Melissa K. Maulding, Heather A. Eicher-Miller, Dennis A. Savaiano, Regan L. Bailey

**Affiliations:** 1 Department of Nutrition Science, Purdue University, West Lafayette, IN 47907, USA; jun24@purdue.edu (S.J.); tvsowmy@gmail.com (S.V.T.); heicherm@purdue.edu (H.A.E.-M.); savaiano@purdue.edu (D.A.S.); 2 Health and Human Sciences Extension, Purdue University, West Lafayette, IN 47907, USA; mmaulding@purdue.edu

**Keywords:** NHANES, SNAP-Education, SNAP, low-income, nutrition, fruit, vegetable, whole grain, diet disparities, Dietary Guidelines

## Abstract

The Supplemental Nutrition Assistance Program-Education (SNAP-Ed) program aims to improve nutritional intakes of low-income individuals (<185% poverty threshold). The objective of this study was to describe the compliance with Dietary Guidelines for Americans (DGA) recommendations for fruits, vegetables, and whole grains among SNAP-Ed eligible (*n* = 3142) and ineligible (*n* = 3168) adult women (19–70 years) nationwide and SNAP-Ed participating women in Indiana (*n* = 2623), using the NHANES 2007–2012 and Indiana SNAP-Ed survey data, respectively. Sensitivity analysis further stratified women by race/ethnicity and by current SNAP participation (<130% poverty threshold). Nationally, lower-income women were less likely to meet the fruit (21% vs. 25%) and vegetable (11% vs. 19%) guidelines than higher-income women, but did not differ on whole grains, which were ~5% regardless of income. The income differences in fruit and vegetable intakes were driven by non-Hispanic whites. Fewer SNAP-Ed-eligible U.S. women met fruit (21% vs. 55%) and whole grain (4% vs. 18%) but did not differ for vegetable recommendations (11% vs. 9%) when compared to Indiana SNAP-Ed women. This same trend was observed among current SNAP participants. Different racial/ethnic group relationships with DGA compliance were found in Indiana compared to the nation. Nevertheless, most low-income women in the U.S. are at risk of not meeting DGA recommendations for fruits (79%), vegetables (89%), and whole grains (96%); SNAP-Ed participants in Indiana had higher compliance with DGA recommendations. Increased consumption of these three critical food groups would improve nutrient density, likely reduce calorie consumption by replacing high calorie choices, and improve fiber intakes.

## 1. Introduction

The Supplemental Nutrition Assistance Program-Education (SNAP-Ed), an educational component of Supplemental Nutrition Assistance Program (SNAP), provides nutrition education and obesity prevention strategies consistent with Dietary Guidelines for Americans (DGA) to improve the nutrition and health of participants [1]. SNAP-Ed is delivered to low-income individuals at or below 185% of poverty-to-income ratio (PIR) with a primary focus on low-income women and is administered at the state-level. Few studies have evaluated the effect of SNAP-Ed on dietary intakes among adults although the program was associated with increased intention to change nutrition-related behaviors among adults [2], and a SNAP-Ed intervention was effective in improving children’s fruit and vegetable intakes [3]. In addition, little is known about dietary intakes of SNAP-Ed participating or eligible women, particularly regarding meeting the Dietary Guidelines for Americans (DGA) policy goals [4], and how state-level program participants compare to the nation.

Fruits, vegetables, and whole grains are important sources of not only essential vitamins and minerals, but also dietary fiber, contributing about 60% of food sources of fiber in the US diet [5]. Fiber has been classified as both a shortfall nutrient and a nutrient of public health concern because there is high prevalence of inadequate intakes across the population, and low intakes are associated with adverse health outcomes [4,6,7,8]. Fiber intake, while low in many segments of the U.S., is of particular concern for women with low incomes [7]. Increasing intakes of these healthy food groups would improve intakes dietary fiber and many other shortfall nutrients and may also help shifting from high calorie choices [4]. Therefore, the objective of this study is to describe compliance with DGA recommendations for fruits, vegetables, and whole grain intakes among SNAP-Ed eligible and ineligible women nationwide from the National Health and Nutrition Examination Survey (NHANES) and from a regional sample of current SNAP-Ed participants in Indiana.

## 2. Materials and Methods

### 2.1. NHANES Participants and Data Collection

NHANES includes a nationally representative sample of the United States, civilian, non-institutionalized population and is conducted by the National Center of Health Statistics, Centers for Disease Control and Prevention [9]. The NHANES protocol was approved by the National Center of Health Statistics Research Ethics Review Board. Participants of this study were selected based on survey year, sex, age, and PIR to facilitate comparison with the sample of Indiana SNAP-Ed participants. The SNAP-Ed eligible U.S. women group included 3142 women aged 19–70 years in low-income households (PIR ≤ 185%) who participated in the NHANES 2007–2012. The SNAP-Ed ineligible U.S. women group consisted of 3168 women aged 19–70 years in higher-income households (PIR > 185%) in the NHANES 2007–2012. PIR is the ratio of family income to poverty guidelines, specific to family size, and is used to determine financial eligibility for most federal nutrition programs, including SNAP (≤130% PIR) [10].

The NHANES protocol includes in home interview where demographic and self-reported health information is collected. Age, education, race/ethnicity, and physical activity were categorized to match the Indiana SNAP-Ed sample survey categories. Age was categorized as 19–30 years, 31–50 years, and 51–70 years. Education level was categorized as less than high school, high school diploma or General Equivalency Diploma (GED), some college or associate degree, and Bachelor’s degree or above. Race/ethnicity was categorized as non-Hispanic White, non-Hispanic Black, and Hispanic and Mexican American (hereafter referred to as White, Black, and Hispanic/Mexican). Other racial/ethnic classifications, including non-Hispanic Asians, were not included in this analysis. Self-reported minutes for walking, bicycling, moderate recreational activities, vigorous recreational activities, moderate-intensity work, and vigorous-intensity work in NHANES data were combined to match the variable for the duration of any type of physical activity in Indiana SNAP-Ed survey data. Physical activity level was categorized as follows: less than 10 min, 10–29 min, 30–60 min, and more than 60 min. Current SNAP participation was limited to NHANES 2011–2012 because this was the only survey cycle to specifically collect this information. 

Following the home interview, participants attended a mobile exam center where a health examination and an in-person 24-h dietary recall were completed. Cup equivalents of fruit and vegetable intakes, and ounce equivalents of whole grain intake reported by each respondent on one-day 24-h dietary recall were obtained from the Food Patterns Equivalents Database (FPED) [11]. Cup- and ounce-equivalents standardize the food and beverage amounts and different forms [4].

### 2.2. Indiana SNAP-Ed Participants and Data Collection

SNAP-Ed in Indiana is directed by Purdue University Health and Human Sciences Cooperative Extension. Indiana SNAP-Ed nutrition education paraprofessionals collected survey data from adults aged ≥ 19 years who participated in SNAP-Ed during 2007 to 2012, before taking any of educational program. Because more than 80% of the total survey respondents (*n* = 3307) were women aged 19–70 years old, our analysis was restricted to women. Therefore, the final Indiana SNAP-Ed participant sample included 2656 adult women aged 19–70 years. The Human Subjects Committee of the Purdue University Institutional Review Board approved all the study protocols and all participants provided written informed consent.

The Indiana SNAP-Ed evaluation survey queried demographics, physical activity level, and food group intakes. All the Indiana SNAP-Ed survey questions were categorical. Race and ethnicity were self-reported and classified to correspond to the national categories [9]. Dietary intake was assessed through a quantity-based (i.e., cup and ounce equivalents) series of questions that ascertained intake of whole fruits, 100% fruit juice, vegetables, and whole grains. Questions were asked as “How much fruit/100% fruit juice/vegetable/whole grain food do you eat?”, for which participants could select from “none/0.5 cup/1 cup/1.5 cups/2 cups/2.5 cups/3 cups or more”. To align with DGA guidelines, whole fruit and 100% fruit juice intake were combined as total fruit intakes. 

### 2.3. Data Analysis

Descriptive statistics for demographic characteristics (i.e., age group, race/ethnicity, and education level) and physical activity level and the percentages of the group meeting the 2015 DGA recommendations for fruits, vegetables, and whole grains were examined for SNAP-Ed eligible (PIR ≤ 185%) and ineligible (PIR > 185%) U.S. women and Indiana SNAP-Ed participating women. The 2015 DGA provides recommendations tailored by daily energy intake [4]. We used DGA guidelines for 1800 kcal/day based on mean energy intakes of SNAP-eligible U.S. women group: 1.5 cups/day of fruit (including whole and dried fruit and 100% fruit juices only), 2.5 cups/day of vegetables, and 3 ounces/day of whole grains [4]. In addition, we calculated the percentages meeting the DGA recommendations stratified by racial/ethnic subgroups and by current SNAP participation subgroups. Comparisons of guideline compliance were made between SNAP-Ed eligible and ineligible U.S. women groups and between Indiana SNAP-Ed participating women group and SNAP-Ed eligible U.S. women group without and with stratification by race/ethnicity or current SNAP participation status (PIR ≤ 130%). Comparisons between stratified groups within Indiana SNAP-Ed participating women group and SNAP-Ed eligible women group were also conducted. Lastly, for sensitivity analysis, we examined compliance with guidelines among Indiana SNAP-Ed participants, controlling for age, race/ethnicity, and education level.

All NHANES estimates were weighted to account for the sampling design to represent the U.S. population. We compared the Indiana SNAP-Ed estimates with the weighted NHANES estimates, and as recommended [12], considered statistical significance only when the 95% confidence intervals did not overlap as no statistical procedures are developed to compare complex sampling frameworks directly with simple sampling frameworks. Statistical comparisons within each regional group were completed using multiple pairwise t tests. Statistical significance was set at *P* < 0.05. All statistical analyses were accomplished with SAS version 9.4 (SAS Institute, Inc., Cary, NC, USA).

## 3. Results

About a half of SNAP-Ed eligible women nationwide were White, whereas about 80% of Indiana SNAP-Ed women and SNAP-Ed ineligible women nationwide were White (Table 1). In both the U.S. and Indiana, most low-income women had no college or higher educational attainment. About a half (53%) of the SNAP-Ed eligible women nationwide reported physical activity less than 30 min a day with 35% reporting more than 60 min per day, while 63% and 14% of Indiana SNAP-Ed women participants reported physical activity less than 30 min a day and more than 60 min per day, respectively. 

**Figure 1:**
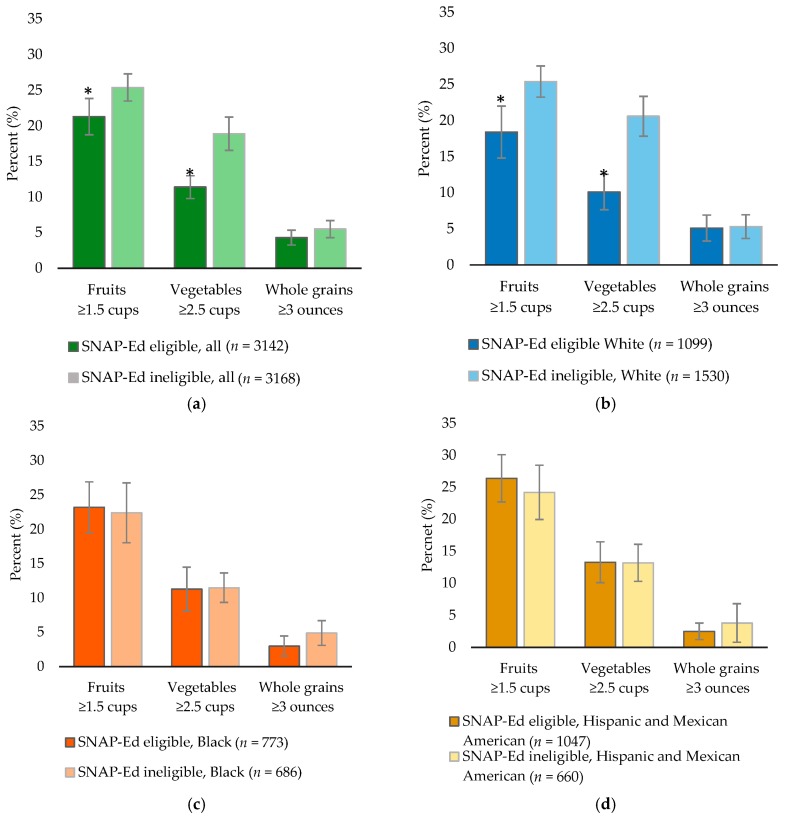
The percentage meeting the Dietary Guidelines for American recommendations of SNAP-Ed eligible and SNAP-Ed ineligible women nationwide, NHANES 2007–2012. (**a**) All (**b**) White (**c**) Black (**d**) Hispanic/Mexican American. Fruit and vegetable intakes are measured in cup equivalents and whole grain intake is measured in ounce equivalents. * Significantly different between SNAP-Ed eligible and SNAP-Ed ineligible women nationwide based on *t*-tests. Error bars indicate 95% confidence intervals.

SNAP-Ed eligible U.S. women had lower compliance with fruit (21% vs. 25%) and vegetable (11% vs. 19%) recommendations than SNAP-Ed ineligible U.S. women, while no differences existed for whole grains (Figure 1). Differences in fruit and vegetable intakes in the nation appear to be largely be driven by income differences in White women, as no differences were noted in Black or Hispanic/Mexican women.

Significantly less SNAP-Ed eligible women nationwide met the DGA recommendations for fruits (21% vs. 55%), and whole grains (4% vs. 18%) than Indiana SNAP-Ed women (Table 2). Compliance with vegetable recommendations was very low (~10%) in both groups and was not significantly different between SNAP-Ed eligible U.S. women and Indiana SNAP-Ed women. To determine if the results are an artifact of the demographic differences, Indiana models were controlled for age, race/ethnicity, and education and a similar pattern of results were obtained (data not shown). Consistently, when stratified by race/ethnicity and by current SNAP participation status (i.e., PIR < 130%), fewer SNAP-Ed eligible women nationwide met fruit and whole grain recommendations than Indiana SNAP-Ed women. 

Within SNAP-Ed eligible U.S. women, White women had lower compliance with fruit recommendation than Black or Hispanic/Mexican women and higher compliance with whole grain recommendation than Hispanic/Mexican women (Table 2). Within the Indiana SNAP-Ed group, Black women had higher compliance with fruit recommendations and lower compliance with whole grains when compared to White or Hispanic/Mexican women. No racial/ethnic differences in vegetable consumption were observed within the national or Indiana group. 

## 4. Discussion

US dietary patterns are not aligned with federal recommendations [13]. Disparities exist for diet quality and intakes of key nutrients and food groups across income gradients [7,14,15,16,17]. We focused the current analysis on low-income women because this is a target group for SNAP-Ed. The results of this study confirmed lower than DGA-recommended intake amounts of fruits, vegetables, and whole grains among low-income women both in the nation and in the state of Indiana. Income differences in compliance with fruit and vegetable recommendations were also confirmed by comparing SNAP-Ed eligible and ineligible women nationwide; this relationship was largely driven by differences in White women, as no differences were noted in either Black or Hispanic/Mexican women.

The food groups examined in this analysis are the key contributors to fiber intake in the U.S. diet. A recent NHANES report described an income gradient for mean whole grain intakes with sexes combined; those in the lowest income category (PIR < 131%) had 10% of total grains that are whole, while higher income categories had 12% (PIR 131–350%) and 17% (PIR > 350%) [18]. However, lower-quality diets are not confined to the lowest income group alone in the United States. Three other recent nationally-representative reports have documented that those in both the lowest and middle-income categories are less likely to meet fruit, vegetable, and whole grain recommendations [16], and also less likely to have usual intakes of shortfall nutrients and fiber aligned with the Dietary Reference Intakes when compared to the highest income group [7,15]. Fruits, vegetables, whole grains, and fiber play an important role in the prevention of chronic diseases [19,20,21,22,23,24,25,26]. Our findings underscore the need for nutrition intervention to improve healthy food choices among all low-income women, but also point to regional and racial/ethnic differences in compliance patterns that may help to inform educational messages for SNAP-Ed.

Previous Behavioral Risk Factor Surveillance System (BRFSS) data, combining men and women, indicate that compliance with fruit and vegetable intake recommendations were lower in Indiana than the national average [27,28]. Because BRFSS data include men and all income levels, the data in our analysis is not directly comparable to BRFSS. Nevertheless, the observed higher total fruit intake in Indiana women was unexpected. This finding should be interpreted with the following aspects or caveats in mind. First, Indiana SNAP-Ed participants who voluntarily decided to participate in SNAP-Ed lessons may have different characteristics compared to eligible nonparticipants. It would be interesting to examine whether nutrition education program participants already have healthier dietary habits [29]. Second, the dietary intakes in Indiana were assessed with a quantitative frequency-based screening method whereas the NHANES collects dietary information through a 24-h dietary recall. Previous comparisons between these 2 methods suggest that the 24-h recall estimates of fruit are lower, while vegetables are similar when compared to frequency-based screening method [30]. This suggest that the use of brief screener in Indiana SNAP-Ed survey could have overestimated fruit intake of Indiana SNAP-Ed women. Finally, there were far fewer Blacks and Hispanics among Indiana SNAP-Ed group than SNAP-Ed eligible U.S. group, which reflects the racial/ethnic composition in Indiana [31]. When controlled for age, race/ethnicity, and education, the estimates from food group intakes were preserved (data not shown). However, racial/ethnic differences within each regional group were noted. Further research should explore the interactive effect of sex, race/ethnicity, and income in adherence to dietary recommendations. Nevertheless, the major strength of this study was that we used at the nationally representative NHANES data to provide national estimates of DGA compliance for all SNAP-Ed eligible women in the U.S. In addition, this study also adds to the paucity of data on dietary intake of SNAP-Ed participants. 

In conclusion, many low-income women were not meeting fruit, vegetable, and whole grain recommendations. Future studies should investigate specific challenges linked with low-income women’s food choices (e.g., cost, transportation, quality, variety, food environment, and societal norms) [32]. In the state of Indiana, SNAP-Ed has been successful at reducing food insecurity in women and families [33,34]; this suggests that SNAP-Ed has the potential to improve diet and health as food insecurity has been directly linked with poor diet quality [35], and suboptimal biomarkers of nutrition status [36,37], and many chronic diseases [38,39]. Two recent studies in California suggested that SNAP-Ed programing is associated with increased fruit and vegetable intakes as larger gains were observed in areas with more SNAP-Ed reach [40,41]. However, in these reports, SNAP-Ed participation was not associated with any significant changes in overall diet quality [40], whereas a study in the U.S. Mountain region found that Expanded Food and Nutrition Education Program, another federally funded nutrition education program, is associated with improved overall diet quality [42]. Therefore, rigorous evaluation of this program is warranted. A need also exists for trials investigating how to best tailor SNAP-Ed programming to enhance diet quality and food security in ethnically diverse populations. SNAP-Ed practitioners in ethnically diverse populations may consider tailoring lessons to be culturally specific for food group intakes [43]. 

## Figures and Tables

**Table 1:** Demographic and lifestyle characteristics of SNAP-Ed participating women in Indiana and SNAP-Ed eligible and ineligible women nationwide, 2007–2012.

	Indiana SNAP-Ed (*n* = 2623) %	SNAP-Ed Eligible Nationwide ^1^ (*n* = 3142) %	SNAP-Ed Ineligible Nationwide ^1^ (*n* = 3168) %
**Age, years**			
19–30	46.2	34.1	18.4
31–50	35.9	39.4	42.0
51–70	17.9	26.5	39.6
**Race/ethnicity ^2^**			
Non-Hispanic White	79.2	51.8	76.9
Non-Hispanic Black	7.2	19.3	8.7
Hispanic and Mexican American	9.8	22.2	8.1
**Education**			
Less than high school	25.2	31.3	7.5
High school diploma or GED	36.8	25.8	17.5
Some college or associate degree	33.3	31.7	34.4
Bachelor’s degree or above	4.9	11.2	40.6
**Physical Activity ^3^**			
Less than 10 min	31.3	41.6	31.9
10–29 min	31.2	9.6	6.7
30–60 min	23.4	13.4	20.6
More than 60 min	14.1	35.4	40.7

Abbreviations: GED, General Equivalency Diploma; SNAP-Ed, Supplemental Nutrition Assistance Program-Education; ^1^ Data were obtained from the NHANES 2007–2012 and weighted to represent the U.S. population; ^2^ Individuals who were identified as ‘other’ for race-ethnicity are not presented, thus the percentages do not add to 100; ^3^ Include all types of physical activity, including walking, bicycling, moderate recreational activities, vigorous recreational activities, moderate-intensity work, and vigorous-intensity work.

**Table 2:** The percentage meeting the Dietary Guidelines for American recommendations, overall and stratified by race/ethnicity and current SNAP participation status, among SNAP-Ed participating women in Indiana and SNAP-Ed eligible women nationwide, 2007–2012 ^1^.

	Indiana SNAP-Ed %	SNAP-Ed Eligible Nationwide ^2^ %
**All**		
*n*	2623	3142
Fruits ≥ 1.5 cups	55.0 (53.1–56.9)	21.3 (18.7–23.8) *
Vegetables ≥ 2.5 cups	9.2 (8.2–10.4)	11.4 (9.8–13.0)
Whole grains ≥ 3 ounces	17.7 (16.3–19.2)	4.3 (3.2–5.3) *
**Non-Hispanic White**		
*n*	2043	1099
Fruits ≥ 1.5 cups	53.1 (50.9–55.2) ^a^	18.4 (14.8–22.0) ^a,^*
Vegetables ≥ 2.5 cups	9.4 (8.2–10.8)	10.1 (7.6–12.5)
Whole grains ≥ 3 ounces	16.5 (14.9–18.2) ^a^	5.1 (3.3–6.9) ^a,^*
**Non-Hispanic Black**		
*n*	186	773
Fruits ≥ 1.5 cups	66.7 (59.4–73.4) ^b^	23.2 (19.5–26.9) ^b,^*
Vegetables ≥ 2.5 cups	10.8 (6.7–16.1)	11.3 (8.1–14.5)
Whole grains ≥ 3 ounces	11.8 (7.6–17.4) ^b^	3.0 (1.6–4.5) ^a,b,^*
**Hispanic and Mexican American**		
*n*	252	1047
Fruits ≥ 1.5 cups	60.3 (54.0–66.4) ^a^	26.4 (22.7–30.1) ^b,^*
Vegetables ≥ 2.5 cups	5.6 (3.1–9.2)	13.3 (10.1–16.5) *
Whole grains ≥ 3 ounces	32.5 (26.8–38.7) ^a^	2.5 (1.2–3.8) ^b,^*
**Currently in SNAP ^3^**		
*n*	1411	433
Fruits ≥ 1.5 cups	54.1 (51.5–56.7)	18.1 (12.8–23.5) *
Vegetables ≥ 2.5 cups	9.5 (8.0–11.1)	10.4 (5.2–15.5)
Whole grains ≥ 3 ounces	16.3 (14.4–18.3)	6.8 (3.1–10.6) *

Abbreviations: SNAP-Ed, Supplemental Nutrition Assistance Program-Education. * Significantly different between Indiana SNAP-Ed and SNAP-Ed eligible women nationwide based on non-overlap in 95% CIs; ^1^ Groups with different letter superscripts (i.e., a or b) denote significantly different within sample. Differences within sample by race/ethnicity and current SNAP participation status were examined with multiple pairwise *t*-tests; ^2^ Fruit and vegetable intakes are measured in cup equivalents and whole grain intake is measured in ounce equivalents. Data were obtained from the NHANES 2007–2012 and weighted to represent the U.S. population; ^3^ Current SNAP participation information is only available for NHANES 2011–2012, so the analysis was restricted to NHANES 2011–2012.
